# *DruGUI* 2.0: mapping protein druggability with probe-based molecular dynamics

**DOI:** 10.1093/bioinformatics/btag429

**Published:** 2026-06-24

**Authors:** Carlos Ventura, Ji Young Lee, Anthony T Bogetti, Anupam Banerjee, Matthew Licht, Ivet Bahar

**Affiliations:** Laufer Center for Physical and Quantitative Biology, Stony Brook University, Stony Brook, NY 11794, United States; Department of Chemistry, College of Arts & Sciences, Stony Brook University, Stony Brook, NY 11794, United States; Laufer Center for Physical and Quantitative Biology, Stony Brook University, Stony Brook, NY 11794, United States; Laufer Center for Physical and Quantitative Biology, Stony Brook University, Stony Brook, NY 11794, United States; Department of Biochemistry and Cell Biology, Renaissance of School of Medicine, Stony Brook University, Stony Brook, NY 11794, United States; Laufer Center for Physical and Quantitative Biology, Stony Brook University, Stony Brook, NY 11794, United States; Department of Biochemistry and Cell Biology, Renaissance of School of Medicine, Stony Brook University, Stony Brook, NY 11794, United States; Laufer Center for Physical and Quantitative Biology, Stony Brook University, Stony Brook, NY 11794, United States; Department of Pharmacological Sciences, Renaissance of School of Medicine, Stony Brook University, Stony Brook, NY 11794, United States; Laufer Center for Physical and Quantitative Biology, Stony Brook University, Stony Brook, NY 11794, United States; Department of Chemistry, College of Arts & Sciences, Stony Brook University, Stony Brook, NY 11794, United States; Department of Biochemistry and Cell Biology, Renaissance of School of Medicine, Stony Brook University, Stony Brook, NY 11794, United States

## Abstract

**Summary:**

We introduce *DruGUI* 2.0, a drug discovery tool for assessing the druggability of proteins, integrated into the *ProDy* application programming interface (API). *DruGUI* 2.0 is developed to facilitate the search for druggable sites while allowing for proteins’ conformational flexibility. Simulations in explicit solvent, with an option to include membrane, are carried out in the presence of probe molecules selected from an expanded library of small molecules containing drug-like fragments. Druggable sites beyond orthosteric sites are identifiable, as well as the probes that show high affinity to bind to those sites. Characterization of the composition and position of the probes helps build pharmacophore models and estimate relative binding affinities. As a Python module with enhanced visualization features, *DruGUI* 2.0 complements, and benefits from, the vast collection of protein sequence, structure, and dynamics analyses modules accessible in *ProDy*. Case studies in the Supplemental Material showcase the utility of *DruGUI* 2.0 applied to both soluble targets and membrane proteins.

**Availability:**

*ProDy* is open-sourced and freely available under MIT License from https://github.com/prody/ProDy. The code version of *DruGUI* 2.0 used for simulations is available on Zenodo : 10.5281/zenodo.20511357.

## 1 Introduction

Drug discovery and development is a lengthy operation, spanning from initial target and hit discovery to FDA approval (Singh *et al*. 2023). It is critical to determine that a target protein is druggable in the early development stage to prevent wasting resources and time, or the so-called attrition effect, at later stages (Nicolaou 2014). To aid in assessing the druggability of targets through computational methods, we introduced *DruGUI* in 2012 (Bakan *et al*. 2012). Using *DruGUI*, a target protein is solvated, ionized, and placed in an aqueous environment containing probes, small organic drug-like fragments, to explore transient binding sites via short molecular dynamics (MD) simulations. Once equilibrium is reached, trajectories are analyzed to identify most probable binding sites, and associated probes. The outputs from *DruGUI* are fed to *Pharmmaker* (Lee *et al*. 2020) to create pharmacophore models for virtual screening.

Despite its broad usage, several factors limited the utility of *DruGUI*: the tool was a VMD (Humphrey *et al*. 1996) plugin applicable to soluble proteins only; it was a standalone Python tool separate from the *ProDy* application programming interface (API) (Zhang *et al*. 2021), thus not usable in conjunction with *ProDy* to take advantage of the vast collection of modules *ProDy* houses for sequence, structure, and dynamics analyses, and molecular simulations; it considered only a handful of probe molecules not representing the chemical diversity of FDA-approved drugs or molecules listed in large libraries of compounds; and it was not applicable to membrane proteins, which are major targets for drug discovery; and finally, the underlying force field and simulation package versions were outdated. *DruGUI* 2.0 addresses these limitations. It is now fully integrated into *ProD*y, requiring VMD only to prepare the protein for simulation, and enabling a comprehensive analysis of system dynamics and a thorough evaluation of the significance of druggable sites. In addition, *DruGUI* 2.0 provides access to 100+ diverse probes, is applicable to membrane proteins, and uses the updated CHARMM36 (Huang and MacKerell 2013) force field as opposed to CHARMM27.

## 2 Description and functionality

### 2.1 Input, interface, and preparation

*DruGUI* 2.0 allows users to prepare the druggability simulation system either with a GUI or a non-GUI Python module. The input files for *DruGUI* 2.0 are Protein Structure File (PSF) and Protein Data Bank (PDB) files as well as the user’s VMD executable. PSF and PDB files can be generated through VMD and other tools like CHARMM-GUI (Yu *et al*. 2012). Once the user provides the necessary files and executable path, a probe composition is selected. The default composition, tested and validated on distinct targets (Lee *et al*. 2019, Sutkeviciute *et al*. 2022, Chi *et al*. 2025, Ventura *et al*. 2025), contains isopropanol (16%), acetamide (14%), acetate (14%), isopropylamine (14%), isobutane (14%), imidazole (14%), and benzene (14%). However, depending on the protein of interest and target site, the user may select any combination from the full list of probes. For example, imidazole was added to the probe list due to its strong affinity to bind the heme group when heme-bound targets such as cytochrome *c* were investigated (Bakan *et al*. 2012).

*DruGUI* 2.0 makes a diverse set of 129 probes accessible to users (as opposed to seven in the original *DruGUI*). This set of probes is based on frequency of occurrences in the ‘approved’ and ‘all’ drug categories in DrugBank (Knox *et al*. 2024), and their availability in the CHARMM General Force Field (Yu *et al*. 2012). They feature a diversity of polar, hydrophobic, negatively and positively charged groups, as well as five- and six-membered rings, the combinations of which provide a comprehensive representation of drug-like molecules. The full list of probes is available in the Supplemental Material and the tutorial page http://www.bahargroup.org/prody/tutorials/drugui2_tutorial/index.html.

**Figure 1:**
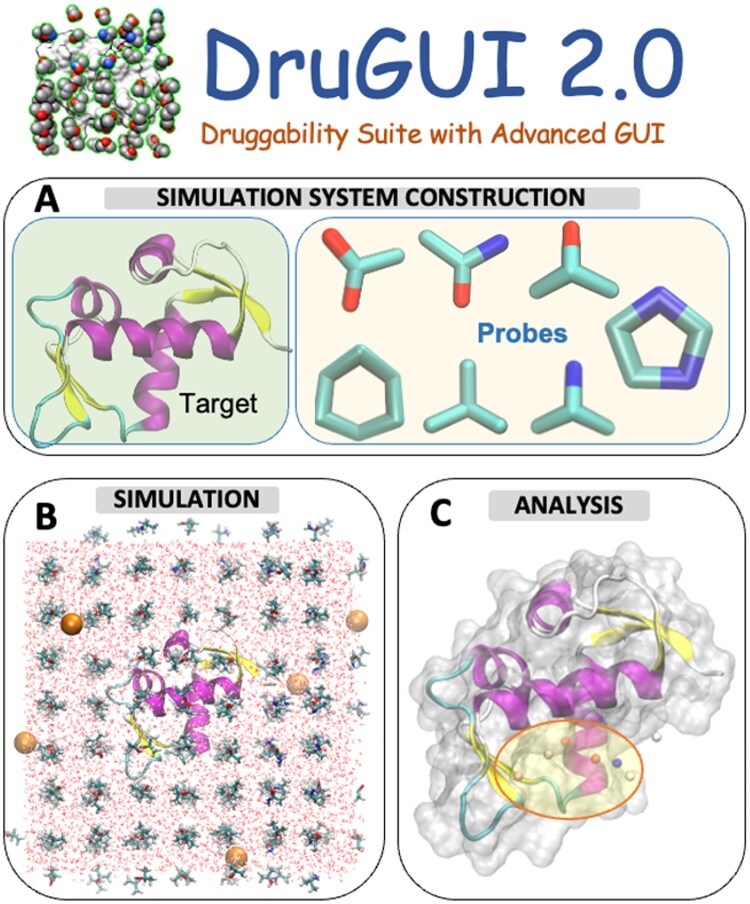
Workflow of *DruGUI* 2.0. (A) *DruGUI* 2.0 uses both PSF and PDB files of a protein of interest (here MDM2) with a probe set and composition to create druggability simulation input files. Here, the default probes of isopropanol, isobutane, imidazole, benzene, acetamide, acetate, and isopropyl amine are shown, with oxygen atoms in *red* and nitrogen atoms in *blue*. (B) A prepared system prior to druggability simulations. The system is solvated, neutralized with ions (*spheres*), and mixed with probes (*sticks*). Simulations are performed with the MD simulation package NAMD3. (C) The trajectories are analyzed to identify ‘hot spots’, the probes binding those sites, and coordinating residues, used for pharmacophore modeling.

Once the target and probes are selected, *DruGUI* 2.0 can be used to (i) solvate the protein, (ii) ionize the water box, and (iii) add an appropriate number of probes based on the padding, boundary, and the presence of a lipid bilayer. Figure 1A shows a target protein, E3 ubiquitin-protein ligase MDM2 (or mouse double minute 2 homolog), and the default probe set adopted for its druggability simulations. Figure 1B shows the simulation box in the final PSF file after preparation.

### 2.2 Simulations

A simulation protocol of NAMD (Phillips *et al*. 2020) is adopted. Minimization of 10,000 (soluble proteins) or 20,000 (membrane proteins) steps is followed by 0.80 ns of annealing, 0.60 ns of equilibration, and multiple production runs of 40 ns for an average size target, which could be extended for larger proteins. A total of 10,000 frames is saved in each run for druggability analysis. The power of *DruGUI* 2.0 is its ability to find druggable sites and solutions with short MD simulations. This is made possible by the annealing step in the simulation protocol. The system’s temperature is maintained at 600 K for a brief time (600 ps), allowing the probes to reach cryptic binding sites that would be inaccessible at normal temperatures.

### 2.3 Druggability analysis

With *DruGUI* 2.0, probe grid and druggability assessment calculations are now performed within *ProDy*. Probe grids are a three-dimensional partitioning of space around the protein into small volume elements (voxels), each used to measure local probe occupancy and density (Seco *et al.* 2009). The user can modify the parameters used for each. Outputs for druggability analysis include occupancy of grids for all probes simulated, PDB files representing various snapshots of the protein and druggable sites, and a summary of each site comprised of their drug-binding affinity, or a lower bound for potential drug binding free energy, and volume. Figure 1C shows MDM2 with its most druggable site (highlighted in the *oval*). This site exhibited a binding free energy of −14.47 kcal/mol with an affinity of 0.028 nM. Notably, this site is the p53 binding pocket of MDM2, and its overlap with the binding probes is shown in the Supplemental Material (Fig. S1, available as supplementary data at *Bioinformatics* online).

A new feature of *DruGUI* 2.0 is the investigation of protein-membrane systems. Figure S2, available as supplementary data at *Bioinformatics* online illustrates the application of *DruGUI* 2.0 to a GPCR, m-opioid receptor. Notably, four major sites are identified: Site 1 is the known G protein binding site; Site 2 overlaps with the substrate (morphine) binding site, evidenced by comparison with a morphine-bound structure of m-opioid receptor; and Sites 3 and 4 are at the membrane-protein interface. GPCRs have allosteric sites at the membrane-protein interface that can be used as potential drug target to modulate function (Hedderich *et al*. 2022). Probes were able to diffuse through the membrane and interact at the membrane-protein interface with the use of a restraining force in the system (Ciancetta *et al*. 2021). Therefore, in addition to capturing known binding sites of substrates or drugs, *DruGUI* 2.0 can detect sites at the membrane-protein interface as well as protein-binding sites, which either overlap with known allosteric sites, or could be targeted by allosteric modulators and further investigated using the modules available in *ProDy*. One such module, *Pharmmaker* (Lee *et al*. 2020) uses *DruGUI* 2.0 results to create pharmacophore models, which are then used for virtual screening of libraries of small molecules.

## 3 Conclusion

*DruGUI* 2.0 is introduced here as a *ProDy* module for assessing the druggability of target proteins by MD simulations in the presence of diverse probe molecules. As illustrated in the Supplementary Material, *DruGUI* 2.0 significantly expands the capability of the originally introduced interface: it yields improved results that better correlate with experiments, and is applicable to membrane proteins, major drug targets, embedded in lipid bilayers of desirable composition. A user-friendly GUI is provided to prepare the simulation system and analyze the trajectories. A non-GUI version is additionally provided for more granular control and automation of system preparation.

As a module integrated into *ProDy*, *DruGUI* 2.0 can be used in conjunction with *ProDy’*s tools for characterizing the structural dynamics of protein targets and better interpreting the results, in addition to constructing pharmacophore models for virtual screening. Recent work has shown that approximately 1/3 of approved drugs bind to hinge sites (Zhang *et al*. 2024). These sites can be identified using the HingeFinder function in *ProDy 2.0*. In addition, *ProDy* 2.0 Essential Site Scanning Analysis module can be used to identify residues predicted to significantly alter global protein dynamics upon ligand binding (Kaynak *et al*. 2020). Together, HingeFinder and ESSA provide complementary criteria for assessing the significance of hotspots identified by *DruGUI 2.0.* Overall, *DruGUI* 2.0 integrated with *ProDy* is expected to serve as a versatile resource for accelerating drug discovery and development.

## Supplementary Material

btag429_Supplementary_Data
