# Supplementary material for: DruGUI 2.0: mapping protein druggability with probe-based molecular dynamics
Source: Bioinformatics. 2026 Jun 24;42(7):btag429. doi: 10.1093/bioinformatics/btag429 (PMC13335477; doi:10.1093/bioinformatics/btag429)
Supplement: btag429_Supplementary_Data [file btag429_supplementary_data.docx]

**SUPPLEMENTARY MATERIAL**

*for*

**DruGUI 2.0: Mapping Protein Druggability with Probe-Based Molecular Dynamics**

**Carlos Ventura^1,2,*^, Ji Young Lee^1^, Anthony T. Bogetti^1,4^, Anupam Banerjee^1,4^, Matthew Licht,^1,3^ and Ivet Bahar^1,2,4,*^**

*^1^Laufer Center for Physical and Quantitative Biology, Stony Brook University, Stony Brook, NY, USA; ^2^Department of Chemistry, College of Arts & Sciences, Stony Brook University, Stony Brook, NY, USA; ^3^Department of Pharmacological Sciences and ^4^Department of Biochemistry and Cell Biology, Renaissance of School of Medicine, Stony Brook University, Stony Brook, NY, USA*

**Supplementary Table**

**Supplementary Table S1. The list of probe molecules accessible in DruGUI 2.0 and their specifications.**

A table of *DruGUI* 2.0 probe molecules in a 3D representation. Residue id and name are based on either their probe representation in CHARMM CGENFF or PBDA (PBDA are custom generated in the original *DruGUI* paper that have slightly different parameters than the CGENFF parameters). Oxygen in atoms are in *red*, nitrogen atoms in *blue*, chlorine atoms in *bright green*, fluorine atoms in *cyan*, iodine atoms in *purple*, bromine atoms in *brown*, and sulfur atoms in *yellow*. Probes are categories as (A) core, (B) hydrophobic, (C) negatively charged, (D) polar, (E) positively charged, (F) six-membered rings, and (G) five-membered rings.

**Supplementary Results**

Two case studies are presented in the Supplementary Results. Case study 1 revisits MDM2, a negative regulator of the p53 tumor suppressor, a test protein in the original study that introduced the druggability simulations (Bakan, et al., 2012). This case study demonstrates how the implementation of new forcefield parameters leads to more accurate results. The second case study consists of a druggability simulation and analysis of the μ**-**opioid receptor in the presence of a POPC membrane, showcasing *DruGUI* 2.0’s ability to assess the druggability of membrane proteins in the presence of the lipid bilayer.

**Case study 1: Enhanced results obtained for MDM2**

Murine double minute 2 homolog (MDM2) is an E3 ubiquitin-protein ligase acting as a negative feedback regulator of p53 (Haupt, et al., 1997). MDM2 acts as a proto-oncogene as p53 is a tumor suppressor protein in humans. The input structure of MDM2 (PDB: 1YCR) (Kussie, et al., 1996) was prepared for druggability simulations with *ProDy*. The original probe composition of isopropanol (70%), acetamide (10%), acetate (10%), and isopropylamine (10%) was adopted. Druggability simulations were performed with NAMD3 (Phillips, et al., 2020) and druggability analysis was performed with *ProDy*.

Simulations are performed with the CHARMM36 force field (Huang and MacKerell, 2013) instead of CHARMM27, the forcefield used in the original *DruGUI*. The most druggable site of MDM2 is the p53 interaction site, as shown in **Figure S1A**. The predicted affinity is 0.028 nM, a ten-fold increase from the previous predicted affinity of 0.30 nM. Similarly, p53 residues F19, W23, and L26 exhibit close overlaps with binding probes shown in **Figure S1B**. The same residues (F19, W23, and L26) were identified to be hot-spot residues in the original study (Bakan, et al., 2012) Not only are the predicted binding affinity and poses of the probes more accurate than those observed in the original study, but the computing time needed for the simulation is drastically reduced. Previously, multiple days were needed for the completion of one run. Using one GPU with NAMD3, the current MDM2 simulation was completed in six hours only.


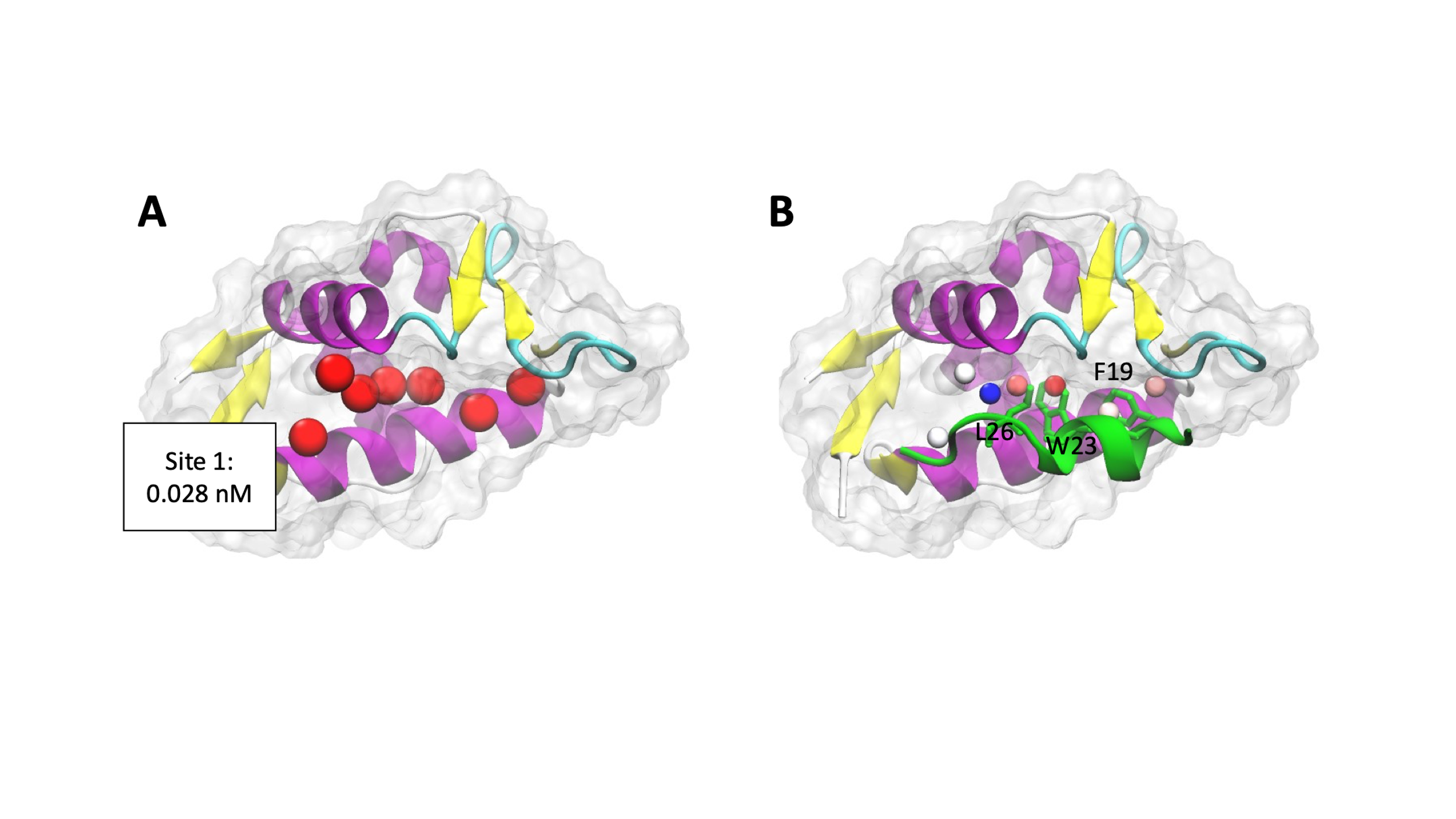


**Figure S1. MDM2 druggability simulation analysis with *ProDy***. (**A**) The most probable druggable site (Site 1) identified in MDM2 (indicated by *red spheres* that correspond to the most frequent binding poses/sites of probe molecules) is the p53 interaction site. In this case, seven isopropanol probes were observed to persistently bind this site with a binding affinity predicted by *DruGUI* 2.0 to be 0.028 nM. (**B**) The diagram shows the overlap between the p53 residues F19, W23, and L26 and the probes binding to Site 1. The probes are color coded by binding affinity from *red* (strongest) to *blue* (weakest).

**Case study 2: Membrane druggability simulation of the Mu Opioid Receptor**

The μ**-**opioid receptors are G protein-coupled receptors (GPCRs) serving as drug targets for treatment of pain (Valentino and Volkow, 2018). GPCRs are important targets for drug discovery as they are targets for 35% of approved drugs (Majumdar, et al., 2024; Sriram and Insel, 2018).

CHARMM-GUI Membrane Builder (Lee, et al., 2019) was used to generate the μ**-**opioid receptor (PDB: 8EF6) (Zhuang, et al., 2022) in a POPC membrane. The protein-membrane complex was prepared in *DruGUI* 2.0 with a probe composition of isopropanol (16%), acetamide (14%), acetate (14%), isopropylamine (14%), isobutane (14%), imidazole (14%), and benzene (14%). **Figure S2A** shows the prepared druggability simulation system prior to simulations. **Figure S2** panels **B-D** show the results from the druggability simulations (performed using NAMD3), analyzed by *ProDy*. Four sites were found to be druggable, shown in **Figure S2B**. Site 1 is the G-protein binding site, Site 2 is the substrate binding site, and Sites 3 and 4 are at the membrane-protein interface. In **Figure S2D**, the guanine nucleotide-binding protein G(i) subunit α1 (Gαᵢ1), shown in *cyan*, overlaps with the probes that occupy the druggable Site 1. Gαᵢ1 was not present in the druggability simulations. Site 2 has a drug-like binding free energy of -12.09 kcal/mol, which is higher than that of Site 1, -14.75 kcal/mol. **Figure S2C** shows how the substrate morphine precisely overlaps with the probes at Site 2. Not all the probes overlap by morphine, indicating that (i) a larger molecule could fit in the active site., or (ii) the additional probes refer to sites temporarily visited by the morphine as it channels to its binding site (Site 2). This type of information may be valuable when making therapeutics for the opioid receptor. Residues L348, C351, L353, and F354 from Gαᵢ1 overlap with the probes in druggable site 1. These residues form important interactions with the opioid receptor to stabilize the opioid receptor-G(i) protein complex (Koehl, et al., 2018; Mafi, et al., 2021). *DruGUI* 2.0 was able to sample sites at the membrane-protein interface of the μ**-**opioid receptor. To find more allosteric sites at the membrane-protein interface, the probe composition can be modified to include probes that can diffuse more easily into the membrane and have more interactions with the interface.

This example shows that *DruGUI* 2.0 is able to (i) detect the substrate binding site of a membrane protein (GPCR) which also serves as a drug binding site, and (ii) unbiasedly identify the protein-protein interaction site (here the G protein binding site) as a site with high avidity to bind a substrate (protein or small molecule).


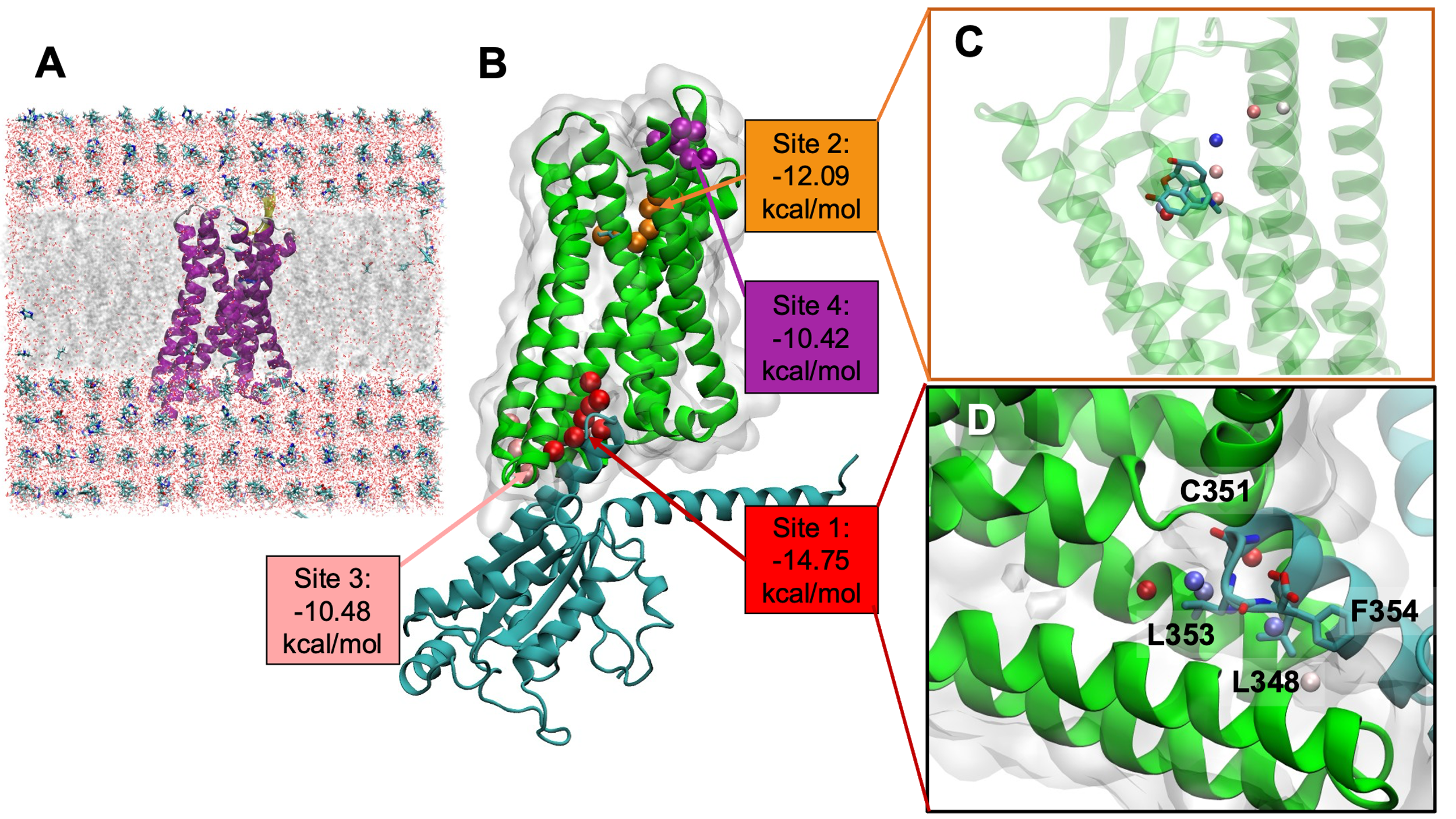


**Figure S2. Results from the druggability simulations of μ-opioid receptor-membrane complex.** Panel **A** displays the side view of simulation system: the target protein (μ-opioid receptor) embedded in a lipid bilayer, surrounded by water containing a uniform distribution of probe molecules. (**B-D**) Results from druggability simulations of μ-opioid receptor. The structure complexed with G protein (*cyan diagram*) is shown here for illustrating the significance of the results; but simulations were carried out for the GPCR (*green ribbon diagram*) only using the atomic coordinates from the PDB structure 8EF6. (**B**) Sites 1, 2, 3, and 4 are the most druggable sites determined from druggability analysis. The substrate binding affinities of Sites 1 and 2 are -14.75 and -12.09 kcal/mol, respectively. Sites 3 and 4 are druggable sites at the membrane-protein interface of the μ-opioid receptor with respective binding affinities of -10.48 and -10.42 kcal/mol. Clusters of seven probes are used to predict druggable sites as their heavy atom count is similar to that of approved drugs. (**C**) Spatial overlap between morphine (not included in the simulations but superposed here for visualization using the resolved structure in the presence of morphine (PDB: 8EF6) and the probes observed in druggability simulations to persistently occupy Site 2. (**D**) Overlap between Gαᵢ1 protein residues with druggable Site 1. L348, C351, L353, and F354 are known to be key residues when forming the complex of the receptor with the G protein.

**Summary**

The utility of *DruGUI* 2.0 is illustrated here using two drastically different systems: a small soluble protein, and a membrane protein embedded in a lipid bilayer. In both systems, which used different probe compositions, druggable sites were identified without bias. *DruGUI* 2.0 has been developed to detect the sites that can serve as binding site for orthosteric or allosteric small molecules. Yet, as shown in the second example, it can also identify sites likely to bind substrate proteins.

Similar computational mapping methods have identified false-positive druggable sites in addition to known orthosteric and allosteric sites (Ghanakota et al., 2019; Raman et al., 2011). Although such false positives were not observed in the two case studies presented here, they may arise in other protein systems. Therefore, *DruGUI 2.0* should be interpreted as a computational mapping and prioritization framework rather than as a definitive classifier of true versus false ligand-binding sites.

In drug discovery applications, regions of interest are sometimes known in advance, such as active sites, allosteric pockets, or protein–protein interaction interfaces. When *DruGUI 2.0* identifies hotspots in these regions, the bound probes can be used directly for downstream applications such as pharmacophore modeling. In cases where binding sites are not predefined, the interpretation may require integrating multiple lines of evidence, including hotspot persistence, estimated interaction energetics, structural context, and orthogonal dynamic analyses available through the *ProDy* API. The integration of *DruGUI 2.0* with *ProDy 2.0* is particularly useful in this regard.

Thus, newly discovered sites from *DruGUI 2.0* should be further analyzed using *ProDy*-guided dynamic and functional-site detection tools and, where possible, validated experimentally. This combined workflow can help prioritize the most biologically relevant and druggable sites while reducing the risk of overinterpreting false-positive hotspots.

The probe composition of isopropanol (16%), acetamide (14%), acetate (14%), isopropylamine (14%), isobutane (14%), imidazole (14%), and benzene (14%) serves as a guideline for druggability simulations for all proteins. If prior information is known about what type of functional group could bind to a target site, the user can modify the probe composition to include probes that complement their protein of interest.

**References**

Bakan, A.*, et al.* Druggability Assessment of Allosteric Proteins by Dynamics Simulations in the Presence of Probe Molecules. *J Chem Theory Comput* 2012;8(7):2435-2447.

Kussie, P.H., *et al*. Structure of MDM2 oncoprotein bound to the p53 suppressor transactivation domain. *Science* 1996;274(5289):948-953

Haupt, Y.*, et al.* Mdm2 promotes the rapid degradation of p53. *Nature* 1997;387(6630):296-299.

Huang, J. and MacKerell, A.D., Jr. CHARMM36 all-atom additive protein force field: validation based on comparison to NMR data. *J Comput Chem* 2013;34(25):2135-2145.

Koehl, A.*, et al.* Structure of the µ-opioid receptor-G(i) protein complex. *Nature* 2018;558(7711):547-552.

Lee, J.*, et al.* CHARMM-GUI Membrane Builder for Complex Biological Membrane Simulations with Glycolipids and Lipoglycans. *J Chem Theory Comput* 2019;15(1):775-786.

Zhuang, Y., *et al*. Molecularr recognition of morphine and fentanyl by the human mu-opioid recepter. *Cell* 2022:185(23):4361-4375.e4319

Mafi, A., Kim, S.K. and Goddard, W.A., 3rd. The G protein-first activation mechanism of opioid receptors by Gi protein and agonists. *QRB Discov* 2021;2:e9.

Majumdar, S.*, et al.* Illuminating the understudied GPCR-ome. *Drug Discov Today* 2024;29(3):103848.

Ghanakota, P., *et al.* Free Energies and Entropies of Binding Sites Identified by MixMD Cosolvent Simulations. *J. Chem. Inf. Model.* 2019;59(5):2035-2024.

Raman, E.P., *et al*. Reproducing Crystal Binding Modes of Ligand Functional Groups Using Site-Identification by Ligand Competitive Saturantion (SILCS*)* Simulations. *J. Chem. Inf. Model.* 2011;51(4):877-896.

Phillips, J.C.*, et al.* Scalable molecular dynamics on CPU and GPU architectures with NAMD. *The Journal of Chemical Physics* 2020;153(4).

Sriram, K. and Insel, P.A. G Protein-Coupled Receptors as Targets for Approved Drugs: How Many Targets and How Many Drugs? *Mol Pharmacol* 2018;93(4):251-258.

Valentino, R.J. and Volkow, N.D. Untangling the complexity of opioid receptor function. *Neuropsychopharmacology* 2018;43(13):2514-2520.
